# Targeting alpha synuclein and amyloid beta by a multifunctional, brain-penetrant dopamine D2/D3 agonist D-520: Potential therapeutic application in Parkinson’s disease with dementia

**DOI:** 10.1038/s41598-019-55830-3

**Published:** 2019-12-23

**Authors:** Deepthi Yedlapudi, Liping Xu, Dan Luo, Gregory B. Marsh, Sokol V. Todi, Aloke K. Dutta

**Affiliations:** 1 Department of Pharmaceutical Sciences, Wayne State University, Detroit, MI 48202 USA; 2 Department of Pharmacology & Department of Neurology, Wayne State University, Detroit, MI 48202 USA

**Keywords:** Target identification, Parkinson's disease

## Abstract

A significant number of people with Parkinson’s disease (PD) develop dementia in addition to cognitive dysfunction and are diagnosed as PD with dementia (PDD). This is characterized by cortical and limbic alpha synuclein (α-syn) accumulation, and high levels of diffuse amyloid beta (Aβ) plaques in the striatum and neocortical areas. In this regard, we evaluated the effect of a brain-penetrant, novel multifunctional dopamine D2/D3 agonist, D-520 on the inhibition of Aβ aggregation and disintegration of α-syn and Aβ aggregates *in vitro* using purified proteins and in a cell culture model that produces intracellular Aβ-induced toxicity. We further evaluated the effect of D-520 in a *Drosophila* model of Aβ_1-42_ toxicity. We report that D-520 inhibits the formation of Aβ aggregates *in vitro* and promotes the disaggregation of both α-syn and Aβ aggregates. Finally, in an *in vivo Drosophila* model of Aβ_1-42_ dependent toxicity, D-520 exhibited efficacy by rescuing fly eyes from retinal degeneration caused by Aβ toxicity. Our data indicate the potential therapeutic applicability of D-520 in addressing motor dysfunction and neuroprotection in PD and PDD, as well as attenuating dementia in people with PDD.

## Introduction

Current estimation indicates that Parkinson’s disease (PD) affects approximately 1–2% of people over the age of 65 years. Although a small subset of population (<10%) acquires PD through genetic mutations, however, it is primarily a sporadic disorder^1,2^. The etiology of PD is not completely understood but recent research advances have provided key insight into some important pathogenic factors such as oxidative stress, aggregation of alpha synuclein (α-syn) protein, and increased iron levels^3–5^. The main symptoms of PD are rigidity, bradykinesia, resting tremor and postural instability along with cognitive and psychiatric complications^6–8^. In the first pharmacological treatment of PD, a combination L-DOPA with a peripheral dopamine decarboxylase inhibitor, dramatically improves the symptoms of the disease by replenishing dopamine in dopamine-depleted basal ganglia of the brain^9,10^. However, motor fluctuations with dyskinesia is associated with long-term use of L-DOPA and also produces a decrease in duration of response to a specific L-DOPA dose^11^. Current treatments for PD suffer from a significant, unmet need since these only address symptomatic aspects of the disease process and do not prevent disease progression.

Lewy bodies (LBs) and Lewy neurites(LNs) are important pathological hallmarks of PD^3^. The surviving neurons in the substantia nigra of PD patients contain LBs. α-Syn which is an aggregation prone presynaptic protein, has been implicated in the pathogenesis of PD^12,13^. It contributes to the formation of LBs and LNs^12,14^.

It is now clear that a subset of people suffering from PD also suffer from dementia, which is classified as Parkinson’s disease with dementia (PDD)^15^. In fact PD, PDD and Lewy Body disease (LBD) are progressive neurodegenerative disorders characterized by degeneration of the nigrostriatal dopaminergic and brain cholinergic pathways producing the cardinal motor symptoms, cognitive decline and dementia typical for these disorders^8,16,17^. There is now substantial evidence, indicating that a high number of people with PD also exhibit pathology and symptoms of the other diseases such as LBD and Alzheimer’s disease (AD)^17,18^. It is well documented that a high number of people with PD exhibit cognitive decline^19^. Increasing evidence indicates that aggregation of α-syn and Aβ, which are the major pathological markers for PD and AD, are closely linked and might contribute to cognitive decline and dementia in PD^20,21^. For example, in a recent transgenic animal model study, accumulation of α -syn and subsequent neuronal deficits were enhanced by β-amyloid peptides^22^. The patients with LBD exhibit Aβ deposition and a significant number of people with Aβ carry LBs^20^. Also, low levels of Aβ in the cerebrospinal fluid are consistently found in PD with cognitive decline and dementia^23^. PD is now recognized to present a more complex clinico-pathological condition rather than just motor dysfunction.

In the amyloid cascade, proteolytic cleavage of Amyloid Precursor Protein (APP) which is a membrane bound protein, generates Aβ peptide. Aβ is generated when APP is cleaved in a two-step proteolytic process by β- and γ-secretases^24,25^. This results in the formation of Aβ_40_ and Aβ_42_. The latter fragment has a higher propensity to form aggregates and is mainly found in amyloid plaques. There is a debate whether intracellular accumulation of smaller, soluble Aβ is an early event in AD, linked to synaptic dysfunction^26,27^. It has been observed that intracellular accumulation of Aβ is much more toxic than extracellular Aβ^28,29^.

**Figure 1:**
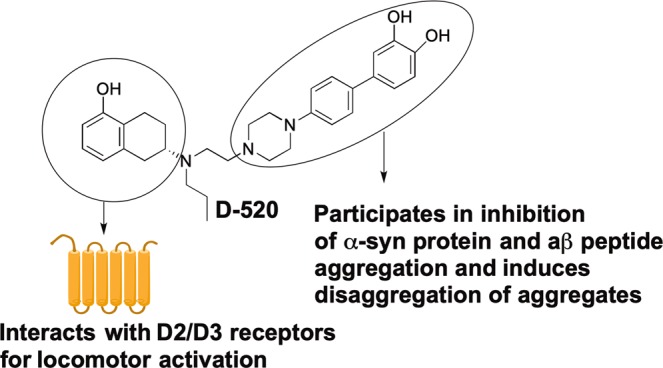
Mode of action for multifunctional activity of D-520.

Our drug discovery research is based on the hypothesis that multifunctional drugs targeting multiple pathogenic factors in PD, or neurodegeneration in general should be more effective and efficacious than a drug targeting only a single target^30,31^. Specifically, we have embarked on the development of novel, multifunctional dopamine D2/D3 agonist molecules as potential symptomatic and disease-modifying treatment agents against PD^32–34^. We have reported several proof-of-concept studies demonstrating the validity of our approach *in vitro* and *in vivo*^35–39^. One such molecule, D2/D3 agonist D-520 (Fig. 1), was recently shown by us to modulate toxicity of α-syn aggregates *in vitro* and *in vivo* independently of dopamine receptor agonist activity^37^. We have also demonstrated that this molecule efficiently crosses the blood brain barrier to stimulate locomotor activity in a PD animal model experiment^40^. In light of its α-syn inhibition activity, we wanted to evaluate the effect of D-520 in disaggregating preformed α-syn aggregates. Furthermore, we sought to evaluate whether D-520 might also decrease aggregation of Aβ peptide and modulate formation and toxicity of Aβ oligomers in human neuroblastoma MC65 cell lines. Finally, in a *Drosophila* model of Aβ_1–42_ dependent toxicity, we wanted to evaluate whether treatment with D-520 could ameliorate such toxicity. Our goal was to assess whether a multifunctional dopamine agonist, like D-520 has the potential to be a treatment agent not only for PD but also for people with PDD. Thus, targeting Aβ peptide in addition to α-syn protein should uniquely qualify D-520 class of molecules as symptomatic and neuroprotective treatment agent for PD and PDD as well as addressing cognitive decline and dementia in PDD.

## Results

### Effect of D-520 on disaggregation of α- synuclein aggregates

**Figure 2:**
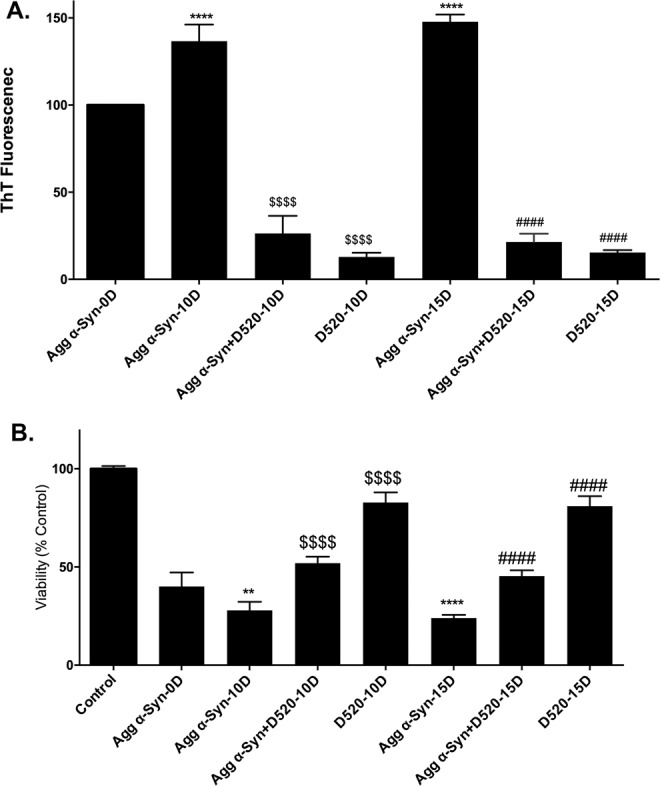
Effect of D-520 on disaggregation of α-synuclein aggregates formed by seeding: (**A**) Aggregates formed by incubating 1.25 mg/mL α-syn with 0.5% PFFs for a period of 30D without shaking were incubated with D-520 for a period of 15 days. The ability of D-520 to dissociate the aggregates was studied by ThT assay at 10D and 15D of incubation. Values are represented in terms of % 0D aggregated synuclein which represents the aggregates collected at 30D from seeding. (**B**) Viability of PC12 cells was measured by MTT assay after 24 h treatment with aggregated synuclein incubated with D-520 collected at 10D and 15D. Values were normalized to control. Data values shown are means ± SD of three independent experiments. One-way ANOVA analysis followed by Tukey’s multiple comparison post hoc test was performed, ^****^p < 0.0001 and ^**^p < 0.01 compared to aggregated Syn-0D,; ^$$$$^*p* ≤ 0.0001 compared to aggregated synuclein-10D and ^####^*p* ≤ 0.0001 compared to aggregated synuclein-15D.

Aggregates of α-syn were generated by seeding as described in the “Methods” section. The aggregates were incubated with D-520 such that the concentration of α-syn was 43.2 μM and that of the compound was 86.45 μM. These incubations were performed at 37 °C without shaking. The inhibition of further aggregate formation and the dissociation of aggregates was confirmed by performing Thioflavin T (ThT) assay of the aliquots collected at days 0, 10 and 15. ThT fluorescence measures the presence of aggregates. The values were normalized with respect to ThT value of aggregated α- syn at day 0 (Agg α-syn-0D) as 100%, which actually represents the aggregates formed from 30 day incubation as described in the “Methods” section. α- syn aggregates continued to aggregate further over the period of 15 days. The increase in ThT fluorescence was 37% and 47% at day 10 and day 15 respectively when compared to aggregated α- syn at day 0 (Fig. 2A). The increase of ThT activity on day 10 and 15 were significant compared to day 0 (Fig. 2A). We observed that D-520 was effective in dissociating the α-syn aggregates significantly from day 10, reaching the peak activity on day 15. When compared to the ThT value of aggregated α-syn alone, D-520 lead to a decrease in aggregation of α-syn by 80% at day 10 and 85% at day 15 respectively. This shows that D-520 is highly effective in dissociating α-syn aggregates. (Fig. 2A).

In order to examine the toxicity of the aggregates formed, PC12 cells were treated with α-syn aggregates at a concentration of 10 µM for a period of 24 h and cell viability was measured by MTT assay. We observed that the toxicity of the aggregates increased with longer incubation. Aggregates collected at 15 days of incubation were most toxic and showed a decrease in viability of 76%, whereas aggregates formed at 10 days showed toxicity of 72%, when compared to control (Fig. 2B). On the other hand, D-520 rescued cells from aggregated α-syn-dependent toxicity. The increase in the viability shown by D-520 when compared to aggregated α-syn is 24% at day 10 and 21% at day 15 respectively. The increase in viability by D-520 was significant at day 10 and day 15 when compared to aggregated α-syn alone (Fig. 2B). Thus, the presence of D-520 could dissociate the α- syn aggregates and prevented them from aggregating further.

### Morphology of α- syn aggregates by transmission electron microscopy

**Figure 3:**
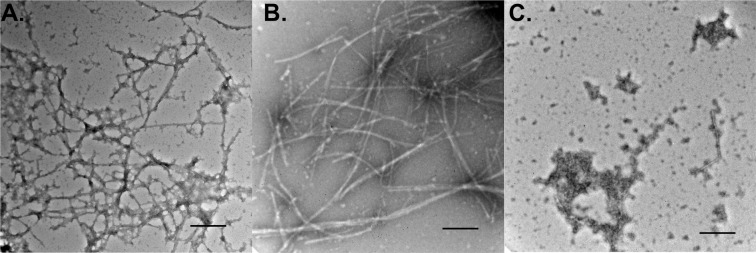
TEM analysis of 30D 0.5% PFF seeded α-syn aggregates. (**A**) Day 0, (**B**) Day 15, (**C**) Day 15 in the presence of D-520; Scale bar = 100 nm.

Transmission Electron Microscopy (TEM) data further corroborated results from ThT and cytotoxicity experiments. As shown in Fig. 3, there was an increase in the extent of α-syn aggregation at 15 days compared to 0 day (Fig. 3B vs. Fig. 3A). This was supported by the increase in ThT activity (Fig. 2A). In this regard, Fig. 3A represents the aggregates generated by seeding α-syn with 0.5% PFF for 30 days (30D)^37^. The morphology of aggregates at 15D is more intertwined than at 0D (Fig. 3A,B). On the other hand, as shown in Fig. 3C, incubation of aggregates with D-520 for 15 days could fully disintegrate the fibrillar aggregates. These results also correlate well with the ThT and toxicity data of α-syn aggregates incubated with D-520. Both ThT and toxicity data showed significant decrease when compared to α-syn aggregates alone (Fig. 2A,B).

### Effect of D-520 on inhibiting oligomerization of Amyloid beta

**Figure 4:**
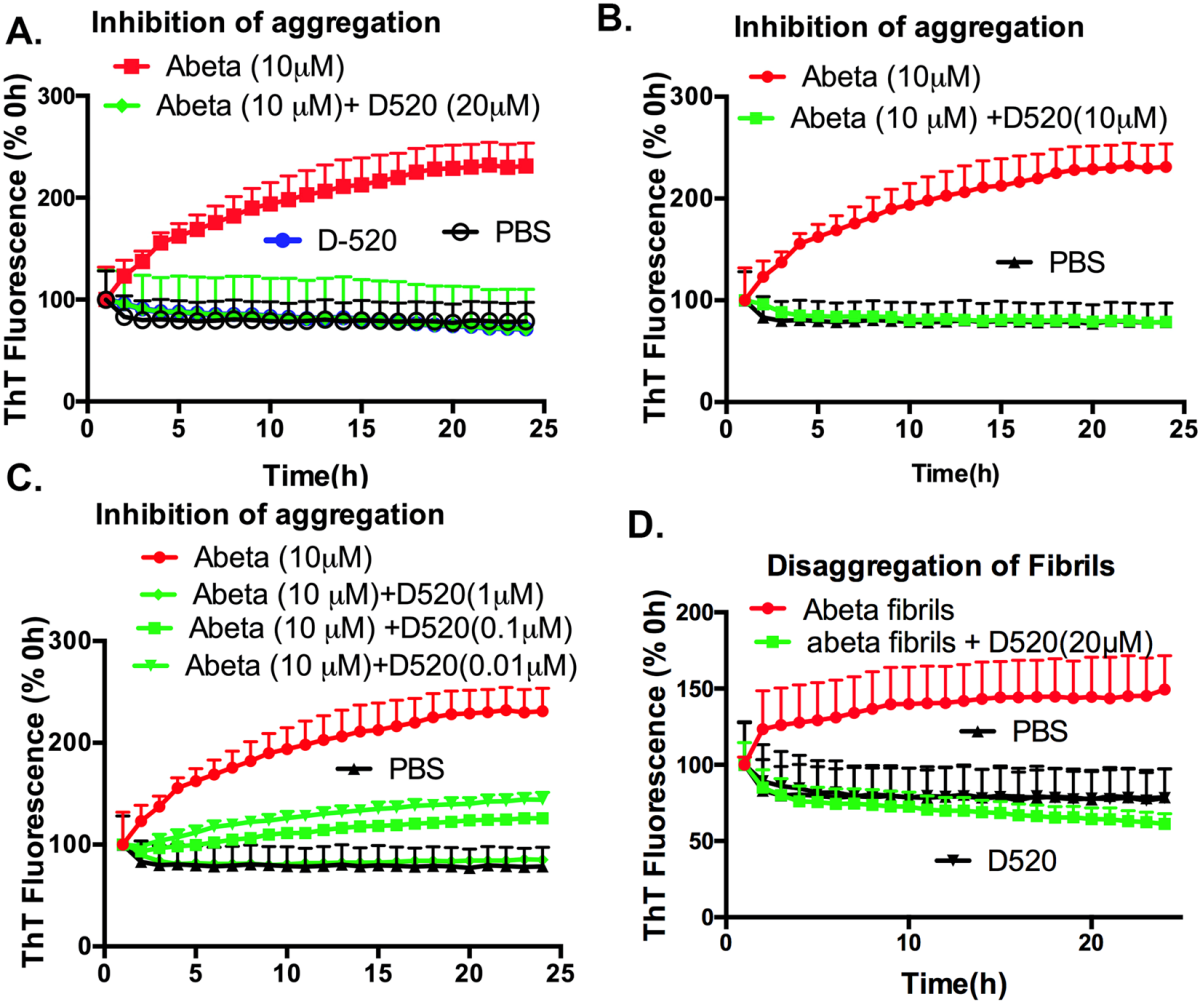
Kinetics on inhibition and dissociation of Aβ_1–42_ oligomer formation by D-520: (**A**–**C**) ThT fluorescence of Aβ_1–42_ (10 μM) with or without incubation with either D-520 (20 μM, Panel: A) or D-520 (10 μM, Panel: B) or D-520 (1 or, 0.1 or 0.01 μM, Panel: C) for 24 h. (**D**) ThT fluorescence of Aβ_1–42_ fibrils (10 μM) with or without incubation with D-520 (20 μM) for 24 h. The ThT fluorescence of Aβ_1–42_ at 0 h was considered as 100%. One-way ANOVA followed by Tukey’s multiple comparison post hoc test, ****P < 0.0001 comparing abeta to abeta + D520 (Panel A-C) and ****P < 0.0001 comparing abeta fibrils to abeta fibril + D520 (Panel D).

Incubation of Aβ_1–42_ at a concentration of 10 µM led to an increase in ThT fluorescence by 131% after 24 h when compared to 0 h (Fig. 4). Kinetics on inhibition of Aβ_1–42_ by D-520 at concentration 20, 10 and 1 μM indicate complete inhibition of the oligomer formation of Aβ (Fig. 4A–C). Inhibition of Aβ_1–42_ was still significant at lower concentrations of D-520, 0.1 and 0.01 μM (Fig. 4C). PBS and D-520 alone did not have any effect on ThT fluorescence.

To evaluate if D-520 can disaggregate pre-formed oligomers as described in the “Methods” section, D-520 was incubated with Aβ aggregates for 24 h and disaggregation was examined by ThT assay. Aβ oligomers incubated alone continued to aggregate over the period of 24 h, as seen by an increase in the ThT fluorescence by 49% when compared to 0 h. However, D-520 was able to induce disaggregation of the formed aggregates and also inhibited the formation of additional Aβ oligomers. This was shown by a decrease in ThT fluorescence by 38% when compared to the Aβ oligomers alone at 24 h (Fig. 4D). Thus, similar to its effect on α-syn, D-520 was able to disassemble Aβ oligomers and prevent further formation of aggregated species of this peptide.

### D-520 protects MC65 cells from Aβ toxicity

**Figure 5:**
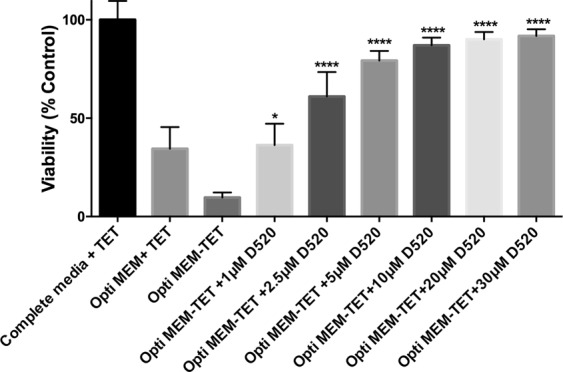
D-520 protects the cells from toxicity caused by Aβ oligomers: MC65 cells were plated in a 96 well plate. Cells received either complete media, or opti MEM with or without Tetracycline. The cells which received media without Tetracycline were treated with different concentrations of D-520 (1–30 μM) for a period of 48 h. Viability was measured by MTT assay. The values were plotted taking control as 100%. Data values shown are means ± SD of three independent experiments. One-way ANOVA analysis followed by Tukey’s multiple comparison post hoc test was performed, *p ≤ 0.1 and ****p ≤ 0.0001 compared to controls grown in complete media with Tetracycline.

Our *in vitro* results, which show that D-520 can prevent or reverse Aβ aggregation, led us to examine whether this modulatory effect has a physiological impact on cell viability when Aβ is present in neuronal cells. We used MC65 cells, which conditionally express Aβ peptides upon removal of tetracycline from the media to examine whether the ability of D-520 to prevent and reverse Aβ aggregation *in vitro* extends to the cellular environment. We observed that the viability of MC65 cells was reduced by 90%, 48 h upon removal of tetracycline from the media. However, treatment with D-520 showed a protective effect by increasing the viability in a concentration-dependent manner. The viabilities shown by 1 μM, 2.5 μM, 5 μM, 10 μM, 20 μM and 30 μM of D-520 were 36%, 61%, 79%, 87%, 90% and 91%, respectively (Fig. 5).

**Figure 6:**
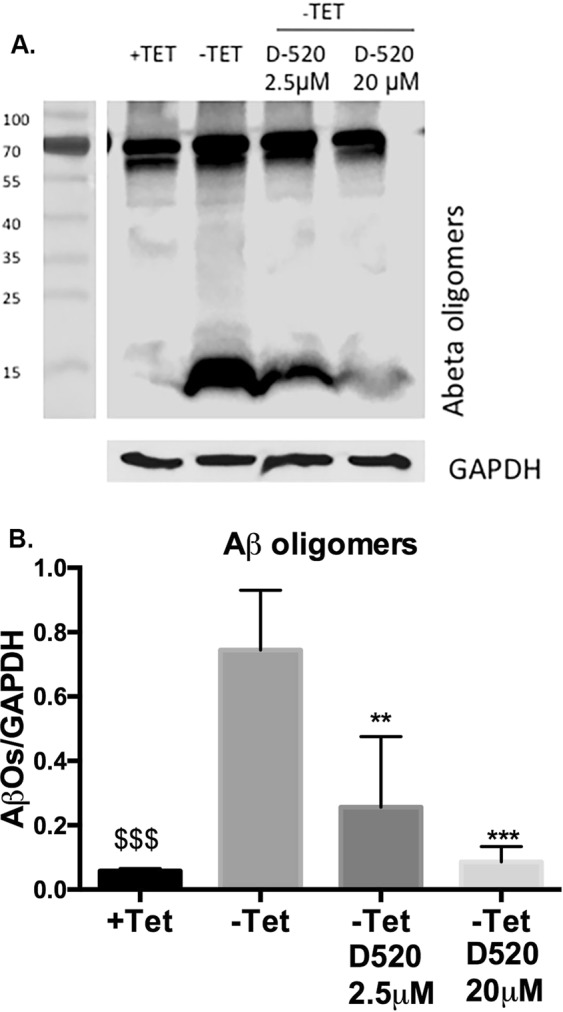
D-520 diminishes the intracellular population of Aβ oligomers: Western blots of extracts from MC65 cell lines that conditionally expresses C99, a 99-residue carboxyl terminal fragment of APP in the absence of tetracycline. C99 is subsequently cleaved by cellular γ-secretase to generate Aβ. The Aβ oligomers are seen when tetracycline is removed from the media. D-520 blocks the formation of Aβ oligomers. Immuno blotting was carried out using the antibody NAB-228 from Cell signalling (upper panel, Panel A)) and GAPDH was used as the loading control. The immuno blot images were quantified in the lower panel (Panel B). The plot shown is representative of 3 replicates. Standard marker as shown in the figure is part of the same gel (see the supplementary material section) and was developed with fluorescence exposure as described in the method section. The results shown is representative of 3 replicates, **p < 0.01, ***p < 0.001 compared to –Tet and $$$p < 0.001 compared to –Tet.

To further understand the mechanisms through which D-520 showed this protective effect, we performed western blot analysis. Western blotting analysis of MC65 cell extracts showed an increase in the levels of Aβ oligomers of varying sizes along with C99 fragments (6–20 KDa range) in cells grown in opti-MEM without tetracycline when compared to the cells grown in complete media with tetracycline. Labeling of higher molecular weight fragments are representative of APP and related fragments (see the original picture of gels in the Supplementary materials) as reported by others^41,42^. However, when cells were treated with D-520, we noticed a decrease in the oligomer formation in a concentration-dependent manner (Fig. 6A). D-520, at a concentration of 20 μM, was more effective when compared to 2.5 μM in the reduction of Aβ oligomers (Fig. 6A,B).

**Figure 7:**
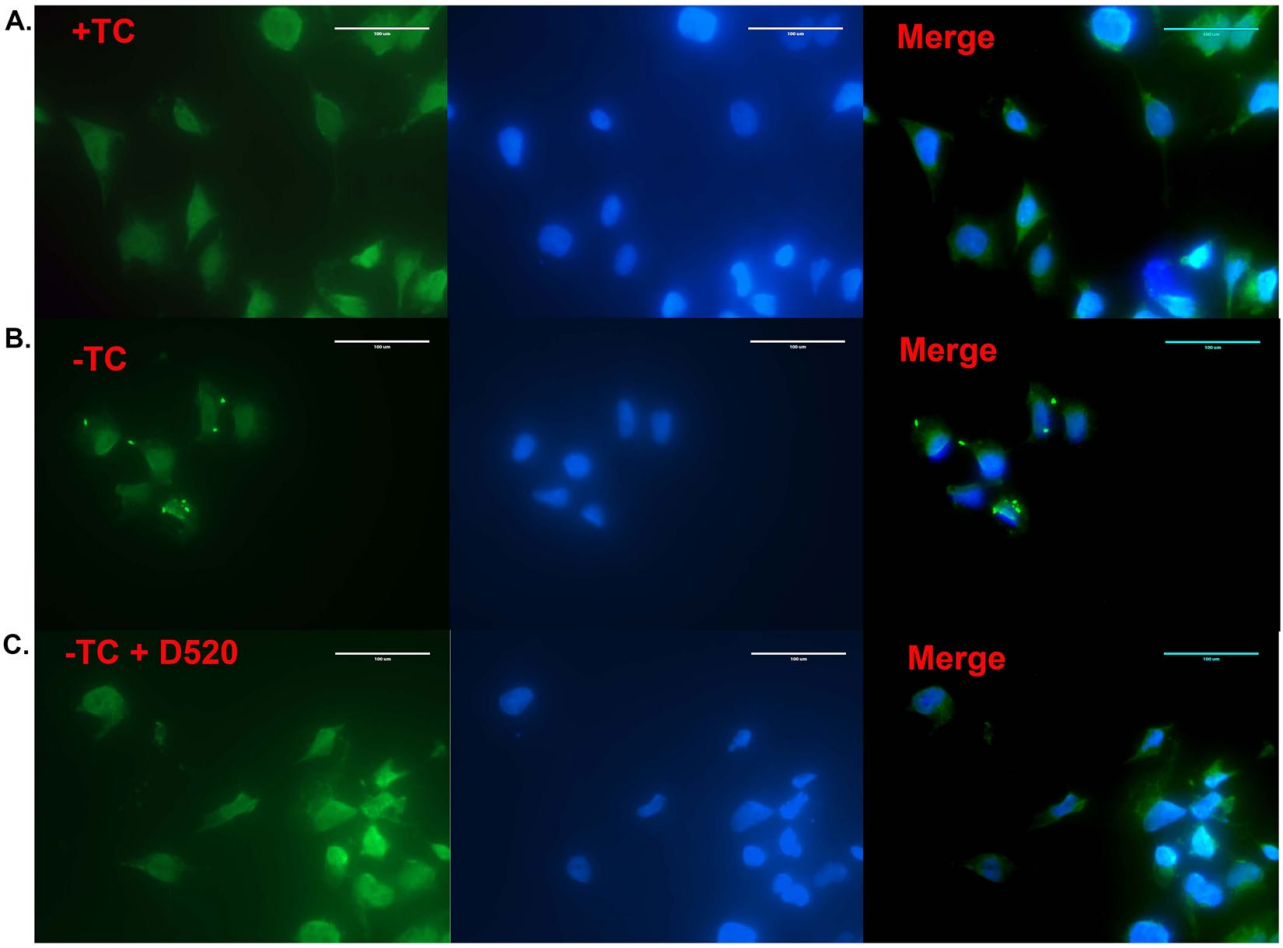
D-520 blocks the appearance of Aβ oligomers in MC65 cells: Immunocytochemistry of MC65 cells from treatment with D-520: MC65 cells were treated with the D-520 at a concentration of 20 μM immediately after the removal of Tetracycline from the media. 48 h after the treatments, cells were fixed and immune fluorescently labeled with Aβ specific antibody (Green) and counter stained with DAPI for nuclear staining. Panel A: control shows the dispersed expression of Aβ. When Tetracycline is removed from the media, it leads to the formation of Aβ oligomers seen as punctate structures with intense green staining (Panel B). However, when the cells are treated with D-520, it leads to the decrease in the Aβ oligomer formation as seen by the even fluorescence throughout the cells (Panel C).

Complementary results on the ability of D-520 to reduce Aβ aggregates in mammalian cells were obtained by using fluorescence based immunocytochemistry method (Fig. 7). We observed the formation of Aβ oligomers, as shown by punctate structures in the cells grown in opti-MEM without tetracycline (Fig. 7, panel B). However, when the cells were treated with D-520 in the absence of tetracycline (Fig. 7, Panel C), the staining was even throughout the cell; no distinct puncta/aggregates were visible. These results indicate that D-520 not only blocked the toxic effects of Aβ oligomers as shown in Fig. 5, but also decreased the formation of oligomeric Aβ species in the MC65 mammalian cell model.

### Suppression of Aβ_1–42_ induced toxicity by D-520 in *Drosophila*

**Figure 8:**
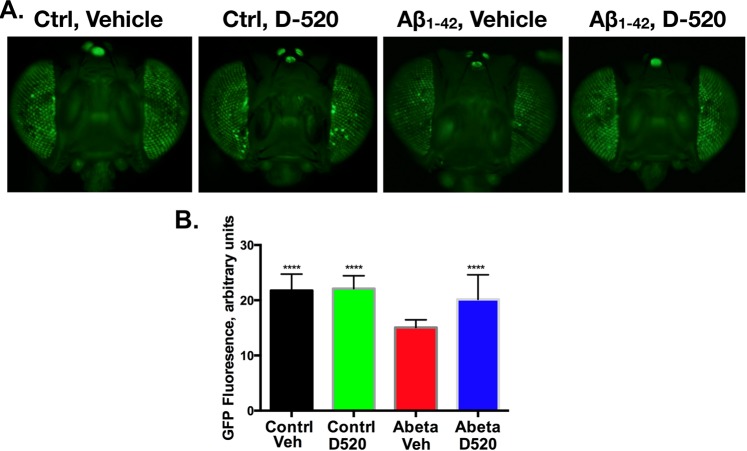
(**A**) Representative images of fly eyes expressing, or not, Aβ_1–42_ in fly eyes, driven by the GMR-Gal4 driver. Flies were 14 days old and were either fed with the vehicle control (ultrapure water) or D-520 (1 mg/mL dissolved in ultrapure water) for 14 days, at which time heads were dissected, imaged and quantified, as summarized in (**B**). Control: GMR-Gal4 driver and UAS-CD8-GFP in the absence of Aβ_1–42_. (**B**) Shown in histograms are GFP fluorescence means −/+ standard deviation. Statistical analyses were conducted with ANOVA. ^***^P < 0.001 comparing columns 1, 2 and 4 to column 3.

We next assessed the ability of D-520 to suppress degeneration in a *Drosophila* model of Aβ_1–42_-dependent toxicity. Expression of Aβ_1–42_ in fly eyes causes degeneration^43^. We had shown before that presence of Aβ_1–42_ in fly eyes leads to a mild loss of fluorescence from membrane-tethered GFP (UAS-CD8-GFP), consistent with loss of fly eye cells^44^. We have utilized this GFP-based technique before to examine and track degeneration caused in fly eyes from various toxic proteins, including polyglutamine disease proteins and α-syn^37,44^. For the present study, we expressed Aβ_1–42_ in fly eyes and fed the flies with either D-520 or its vehicle (ultrapure water) for 14 days after the flies eclosed as adults from their pupal case. As shown in Fig. 8, Aβ_1–42_-expressing flies treated with vehicle led to reduced fluorescence compared to control non-Aβ_1–42_ flies. However, D-520 significantly suppressed the reduction of GFP fluorescence in the fly eyes that was caused by Aβ_1–42_. We conclude that D-520 protects fly eyes from toxicity caused by Aβ_1–42_.

## Discussion

Current treatment of PD mainly focuses on the symptomatic aspects of the disease process. L-Dopa non-responsive dementia is one of the prominent features associated with PD, which occurs in a significant number of the PD patients. Current treatment of dementia in PD focuses on cholinesterase inhibitors and does not provide any disease modification^45^. There is an urgent need for the development of disease modifying treatment agents for PD and PDD.

In this context, we reported here the pharmacological characterization of one of our recent potent dopamine D2/D3 agonists, D-520, for its interaction with α-syn and Aβ. We have shown in our recent reports that D-520 potently inhibits the aggregation of α -syn and modulates toxicity of α -syn aggregates^37,40^. Of additional importance, the modulatory effect of D-520 on α-syn aggregation and toxicity was corroborated in an *in vivo Drosophila* synucleinopathy model, where fly eyes express α-syn. D-520 was shown to reverse the toxicity of α-syn in the fly eyes robustly^37^. In this current report, we have further characterized the effect of D-520 in disaggregating the pre-formed aggregates of α-syn. Since both α-syn and Aβ belong to amyloidogenic proteins, we thought that it is logical to explore any possible interaction of D-520 with Aβ peptides.

Efficacy of D-520 on disintegrating α-syn aggregates was indicated by a decrease in ThT fluorescence right from Day 10 and the lowest ThT fluorescence was observed on Day 15 (Fig. 2). The ThT fluorescence data correlated well with the cell viability data. At Day 15, D-520 showed the maximum protection evident by an increase in the viability (Fig. 2B). At Day10 and Day15, D-520 was clearly protective as it accelerated the dissociation of aggregates, evident from the reduction in ThT fluorescence (Fig. 2A) and morphology of dissociated aggregates (Fig. 3C). These results are encouraging since alteration of toxicity of existing aggregates in the PD brain is a part of neuro-restorative therapeutic strategy.

Similar to the effect of D-520 on α-syn, dose-dependent interaction of D-520 with Aβ_1–42_ peptide clearly indicated a significant effect in inhibiting aggregation in a time-dependent manner, as indicated by a decrease in ThT fluorescence (Fig. 4A–C). In this regard, lower nanomolar doses (10 and 100 nM) of D-520 could still significantly inhibit aggregation of 10 μM Aβ_1–42_ peptide. Given *in vitro* functional activity and *in vivo* efficacy of D-520, this demonstrates that the dual activity of D-520 at dopamine receptors and Aβ_1–42_ could be achieved at a similar therapeutic dose range^40^. Additionally, D-520 decreased ThT fluorescence of preformed Aβ aggregates in a time-dependent manner, reflecting its effect in disrupting structure of aggregates (Fig. 4D). The similarity of interaction of D-520 towards α-syn and Aβ_1–42_ peptide can possibly be due to the fact that both α-syn and Aβ_1–42_ belong to the amyloidogenic class of molecules. Also, in the literature, phenolic compounds have been shown to interact with both of them^46–48^.

In order to firmly establish interaction of D-520 with Aβ peptides in a cellular environment, we carried out our studies with human neuroblastoma MC65 cells, which in the absence of transgene suppressor, tetracycline, produce toxic carboxyl-terminal 99 residues of the Aβ precursor protein leading to generation of Aβ peptides after proteolysis by cellular γ-secretase^41,42^. As shown in Fig. 5, D-520 in the absence of tetracycline rescued MC65 cells from toxicity of Aβ peptides dose dependently, which correlated with our results from inhibition of Aβ_1–42_ shown in Fig. 4A–C. In order to further probe into its interaction with Aβ peptides at a molecular level, western blot studies were carried out to detect the level of Aβ peptides under various treatment conditions with MC65 cells. As expected, we observed a band of Aβ oligomers in the absence of tetracycline which resulted from C-99 fragment (Fig. 6A). Dose dependent increase in cell viability (Fig. 5) was further corroborated by dose dependent reduction of signals of Aβ oligomers and C-99 fragment as detected by the antibody (Fig. 6A,B). These biochemical results were additionally supported by our immunocytochemistry study which showed formation of punctate shaped aggregates in the MC65 cells deprived of tetracycline (Fig. 7B). However, these well-defined oligomeric structures were all together absent in the cells treated with D-520 in the absence of tetracycline (Fig. 7C). This result provides yet another strong evidence that D-520 prevents the formation of Aβ aggregates in the mammalian cellular environment.

To examine whether the protective effect of D-520 against Aβ_1–42_ toxicity in cultured cells can be extended *in vivo*, we used a *Drosophila* Aβ_1–42_-dependent toxicity model. As shown in Fig. 8, D-520 suppressed toxicity from Aβ_1–42_ in fly eyes, compared to the control group of flies, which expressed Aβ, but were not fed D-520. The strong correlation between *in vitro* protein and cell data with our *in vivo* results validates robust interaction of D-520 with Aβ peptides under different environments.

In conclusion, our current work demonstrates that D-520, a potent dopamine D2/D3 agonist that penetrates well into the brain, can inhibit the aggregation and dissociate the aggregates of both amyloidogenic proteins, α-syn and Aβ_1–42_. Collectively, our data indicate that D-520 has the potential to provide neuroprotection and prevent the onset of dementia in PD patients while addressing motor dysfunction as well as mitigating the severity of existing dementia in PDD. Furthermore, our data indicate that engagement of D-520 with these different targets could be achieved within an acceptable dose range, avoiding the potential for high dose related toxicity. Further future studies in additional models will uncover its potential application in the clinic.

## Materials and Methods

### Cell culture

PC12 Adh (ATCC CRL1721.1) cells, a rat adrenal pheochromocytoma cell lines, were purchased from ATCC. RPMI 1640, heat-inactivated horse serum, fetal bovine serum, penicillin-streptomycin, and trypsin were purchased from Gibco. PC12 cells were cultured in RPMI 1640 medium supplemented with 10% heat-inactivated horse serum, 5% fetal bovine serum, and 1% Penicillin Streptomycin (100×, 10,000 Units/ml penicillin, 10,000 g/ml streptomycin) at 37 °C in 5% CO_2_ atmosphere. PC12 cells between the passage numbers 5–15 were used for the experiments.

### Purification of α-syn

pET28 containing α-syn was a generous gift from Dr. Chad M. Rienstra from University of Illinois. α-syn purification was carried out according to Huang *et al*.^49^.

### Generation of α-syn fibrils

Lyophilized α-syn was dissolved in sterile PBS and filtered through a 0.2 μm filter to remove any pre-formed aggregates. α-syn fibrils were generated by incubating purified α-syn at 5 mg/mL at 37 °C under constant agitation at 1000 rpm in a Thermomix R shaker (Eppendorf, Hamburg, Germany) for a period of 5 days. The fibrils generated were then aliquoted and stored at −80 °C until use^50^.

### Generation of α-syn aggregates by seeding

All samples were prepared in PBS. Lyophilized α-syn was dissolved in sterile PBS and was filtered through a 0.2 μm filter to remove any pre-formed aggregates. α-syn at 1.25 mg/mL (86.45 μM) was seeded with 0.5% v/v of preformed fibrils (PFFs) at 37 °C without agitation for a period of 30 days as described by us earlier^37^. α-syn aggregates collected at 30D were used for the subsequent experiments. The formation of fibrils was confirmed by ThT assay.

### Effect of D-520 upon incubation with preformed α-syn aggregates

α-syn aggregates collected from 30D seeding were incubated with compound, D-520 at twice the molar concentration of aggregated syn at 37 °C without agitation for a period of 15 days. Briefly, a stock solution of D-520 at a concentration of 172.9 μM was mixed with equal volume of aggregated α-syn at a concentration of 86.45 μM. This leads to a final concentration of 86.45 μM for D-520 and 43.2 μM for α-syn. Aliquots were collected on day 0, day 10 and day 15. The effect of treatment of D-520 on aggregates was studied by ThT assay. Control group contained D-520 (86.45 mM) which was also incubated at 37 °C and aliquots were collected at different time points for analysis.

### Effect of D-520 from Thioflavin T assay

The dissociation or disaggregation of the fibrils by the compound D-520 was confirmed by ThT assay. 10 μL of the protein was mixed with 10 μL of 40 μM ThT in a black 384-well solid bottom plate (Corning), and the Synergy Hybrid (BioTek) H1 fluorescence microplate reader was used to measure the fluorescence at 440 nm excitation and 485 nm emission wavelength with auto sensitivity mode.

### Evaluation of cytotoxicity of PFF-seeded extracellular α-syn aggregates incubated with D-520

In a 96-wel plate PC12 cells were seeded at 17000 cells/well density. After 24 h the cells were treated with α-syn aggregates or α-syn aggregates incubated with D-520 such that the final concentration of α-syn was 10 μM and that of the drug was 20 μM. The cell viability was measured after 24 h by MTT assay. Next MTT at 5 mg/mL was added to the cells. After incubation for a period of 3 h, the formazan crystals were solubilized in 100 μL of 1:1 mixture of DMSO/Methanol and the absorbance was measured at 570 and 690 nm using an Epoch microplate reader (BioTek, Winooski, VT). Background-corrected values (570−690 nm) were used to plot the graph. Data from at least three experiments were analyzed using GraphPad software (version 6, San Diego, CA).

### Transmission electron microscopy

An aliquot of α-syn samples (4 μL) was adsorbed onto a Formvar-coated, carbon-stabilized copper grid (400 mesh) for 4 min. Distilled water was used twice to rinse the grid briefly followed by negatively stained with 2% aqueous uranyl acetate, air-dried, and examined with a JEOL (JEM 2010) transmission electron microscope at an accelerating voltage of 200 kV and 80,000 or 120,000 magnification.

### Amyloid β_1–42_

Amyloid β_1–42_ ultrapure, TFA (catalog number: A-1002-1) from rPeptide was used for all the studies. Peptides were solubilized as described previously^51^. Briefly, Amyloid β 1–42 peptide was dissolved in 500 μl of HFIP in a 1.5 ml eppendorf microfuge tube and was left in the fume hood until all the HFIP had evaporated. The tube was then placed in a SpeedVac rotary to remove any remaining HFIP. The peptide was then dissolved in DMSO and aliquots were stored at −80 °C until use.

### Effect of D-520 on inhibition of Aβ_1–42_ oligomerization

10 μM of Aβ_1–42_ was incubated with 20–0.01 μM of the compound at 37 °C. All the solutions were made in sterile PBS. ThT at a concentration of 1 μM was added to the reaction mixtures. Aβ_1–42_ alone was considered as positive control. PBS and drug alone were also used as control to monitor their effect on ThT signals. The samples were loaded onto a 96 well black plate and sealed with a film in order to prevent evaporation. Fluorescence readings at 450 nm excitation and 480 nm emission were taken every 1 h over a period of 24 h.

### Effect of D-520 on dissociation of Aβ_1–42_ oligomers

Aβ_1–42_ oligomers were made at a concentration of 10 μM. Briefly, Aβ_1–42_ was incubated at 37 °C with 1 μM ThT in PBS for a period of 24 h. D-520 was subsequently added to oligomers such that its concentration in solution was 20 μM. Fluorescence readings at 450 nm excitation and 480 nm emission were taken every 1 h over a period of 24 h. The ThT values with respect to time were plotted.

### Cell culture models exhibiting intraneuronal Aβ oligomers

We used MC65 (human neuroblastoma cell lines) which shows conditional expression of the carboxyl-terminal 99 residues of the Aβ precursor protein (APP-C99). Aβ is generated after proteolysis by cellular γ-secretase. Removal of the transgene suppressor tetracycline (TC) from the media induces this process as described previously^52^.

Cytotoxicity was determined after 48 h by MTT [3-(4,5-dimethylthiazol-2yl)-2,5-diphenyltetrazolium bromide] assay. Briefly, MC65 cells were plated at a density of 2 × 10^4^ cells/ml in a 96 well plate in complete media containing 1 μg/ml tetracycline. 48 h later, media was changed and controls received either complete media or opti MEM with 1 μg/ml tetracyclin. Aβ oligomerization was induced by adding opti MEM without tetracyclin. To test the efficacy of D-520 towards inhibition of Aβ oligomerization, D-520 was added at different concentrations ranging from 1–30 µM immediately after the removal of tetracycline. D-520 used for these treatments was prepared by incubating it at a concentration of 240 μM in PBS for a period of 6 days followed by dilution to appropriate concentrations described above. Followed by 48 h of treatment, MTT at 5 mg/mL was added to the cells. The volume of MTT solution was 1/10th the volume of the media. After incubation with MTT solution for a period of 3 h, the formazan crystals were solubilized in 100 μL of 1:1 mixture of DMSO/Methanol and the absorbance was measured at 570 and 690 nm using an Epoch microplate reader (BioTek, Winooski, VT). Background-corrected values (570−690 nm) were used to plot the graph. Data from at least three experiments were analyzed using GraphPad software (version 6, San Diego, CA).

### Western blot analysis of inhibition of Aβ oligomer formation by D-520

MC65 cells were harvested for protein extraction 24 h after the treatments. The treatment conditions include: control (complete media with tetracycline), opti MEM with tetracycline, opti MEM without tetracycline, cells treated with either 2.5 μM or 20 μM D-520, prepared in PBS after shaking for six days, in opti MEM without tetracycline media. RIPA lysis buffer was used to lyse the cells in the presence of protease inhibitor cocktail. Protein estimation of the lysates was carried out by BCA method. 40 μg of the proteins were resolved on a 4–20% gradient gel (Biorad). Proteins were transferred onto Nitrocellulose membrane. After blocking the membranes in 5% milk, the membranes were incubated with anti amyloid antibody (cat no: NAB 228 from Cell signaling) at a concentration of 1:1000 overnight at 4 °C. Following this, the blot was washed in TBST three times for 5 minutes each. The blot was then incubated with anti- mouse antibody at a concentration of 1:5000 in 1% non-fat dry milk in TBST for 2 h at room temperature. The image was visualized using ECL-Plus reagent (Perkin Elmer, Waltham, MA, USA) and ImageQuant LAS 4000 imager (GE Healthcare Biosciences, Pittsburgh, PA, USA). Densitometric analysis was performed using ImageJ software. Marker which was part of the same gel, was developed under fluorescence exposure to obtain clearer signals where as Aβ was developed with chemiluminescence method.

### Immuno-florescence for studying Aβ oligomer formation

Immuno-fluorescent labeling of Aβ oligomers in cultured MC65 cells was conducted using the anti amyloid beta antibody listed in the prior section. Briefly, MC65 cells were plated in a 6 well plate in complete media. The cells were allowed to attach for a period of 48 h. The media was then changed to either complete media with Tetracycline or opti MEM without Tetracycline or opti MEM without Tetracycline and with solution of D-520 (20 μM) prepared after six days of shaking. 48 h following the treatments, the cells were fixed in 4% paraforamaldehyde in PBS for 15 min at room temperature. Blocking was done by incubating the cells in 5% BSA in PBS for 1 h at room temperature. The cells were subsequently incubated in anti-amyloid oligomers antibody (AB9234, Millipore) at a 1:1000 dilution at 4 °C overnight. Cells wells were next washed thrice with PBS for five minutes each to remove any unbound primary antibody. The cells were then incubated with anti-mouse alexa fluor 488 at a dilution of 1:500 for 1 h at room temperature. Any unbound secondary antibody was removed by washing the plates thrice with PBS. The cells were then imaged using an Olympus fluorescence microscope at 40X magnification.

### *Drosophila* assays

Fly crosses were conducted and maintained at 25 °C, ~60% humidity in diurnally controlled environments. Crosses were conducted in standard cornmeal media. On the day that adults eclosed from the pupal case, they were switched to instant fly media supplemented with vehicle (ultrapure water) or with final 1 mg/mL D-520 dissolved in ultrapure water. Flies were maintained in media for 14 days, with food changed every 3–4 days. On day 14, fly heads were dissected and GFP fluorescence was examined and photographed with an Olympus BX53 microscope. Fluorescence from fly eyes was then quantified and tabulated as described before^37,44^. Aβ_1–42_ stock was #32038 from Bloomington Stock center. Ctrl flies had the GMR-Gal4 driver and UAS-mCD8-GFP on the w^1118^ background. GMR-Gal4 was #8121 from Bloomington stock center and UAS-CD8-GFP was stock #5137 from Bloomington stock center. UAS-mCD8-GFP and GMR-Gal4 were maintained as a trans-heterozygous stock to cross to w^1118^ or UAS-Aβ_1–42_.

### Statistical analysis

Statistical analyses were performed using GraphPad Prism Version 6 (GraphPad Software, San Diego, CA, USA). We analyzed the data by One-way analysis of variance (ANOVA) followed by Tukey’s multiple comparison post hoc test.

### Ethical approval

As described in the Method section, our animal use protocol (Approved Protocol # 16-03-065) was approved by the Institutional Animal Care and Use Committee at Wayne State University.

## Supplementary information

Supplementry Information
