# Supplementary material for: Targeting alpha synuclein and amyloid beta by a multifunctional, brain-penetrant dopamine D2/D3 agonist D-520: Potential therapeutic application in Parkinson’s disease with dementia
Source: Sci Rep. 2019 Dec 23;9:19648. doi: 10.1038/s41598-019-55830-3 (PMC6927976; doi:10.1038/s41598-019-55830-3)
Supplement: Supplementary file 1 — Supplementry Information [file 41598_2019_55830_MOESM1_ESM.docx]

**Supplementary Materials**

**Targeting alpha synuclein and amyloid beta by a multifunctional brain penetrant dopamine D2/D3 agonist D-520: Potential therapeutic application in Parkinson’s disease with dementia.**

**Deepthi Yedlapudi,^1^ Liping Xu,^1^ Dan Luo,^1^ Gregory B. Marsh,^2^ Sokol V. Todi^2^, Aloke K. Dutta,^1^**

**Western blot analysis of inhibition of Aβ oligomer formation by D-520 in MC65 cells:**

**A.**

**
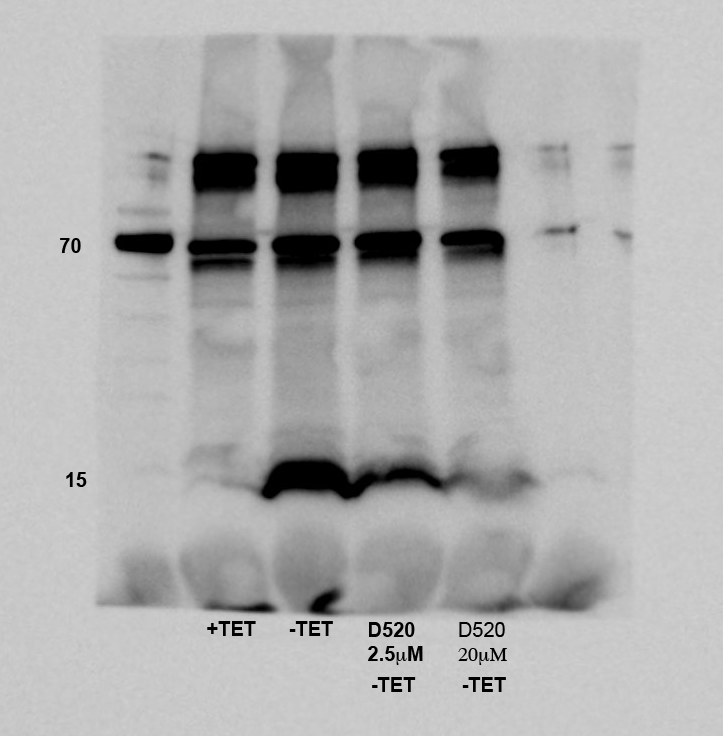
**

**B.**


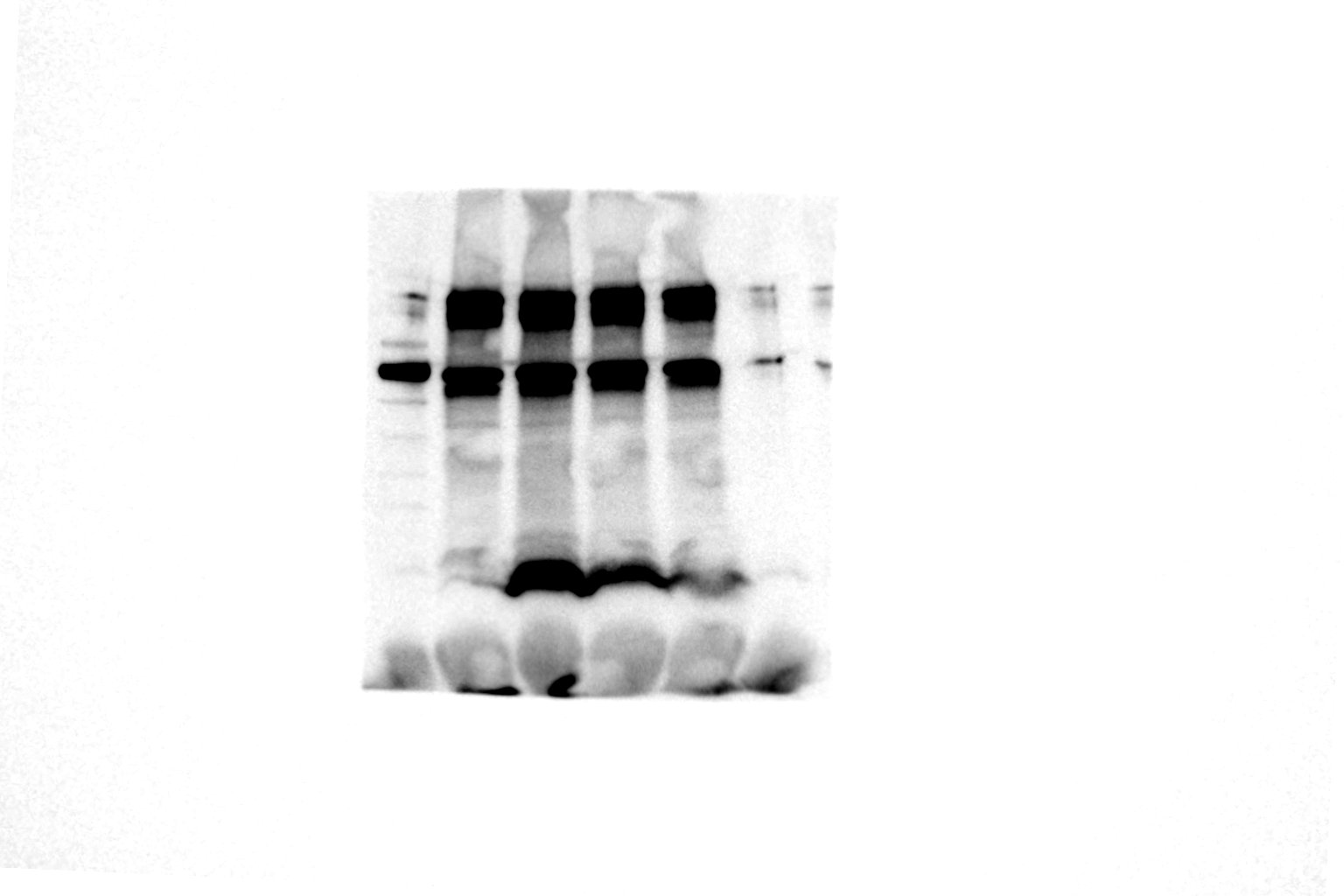


Figure S1: Representative figures of gels from two different exposures. Figure A and B represent two different exposures. The gel was developed by chemiluminescence method.


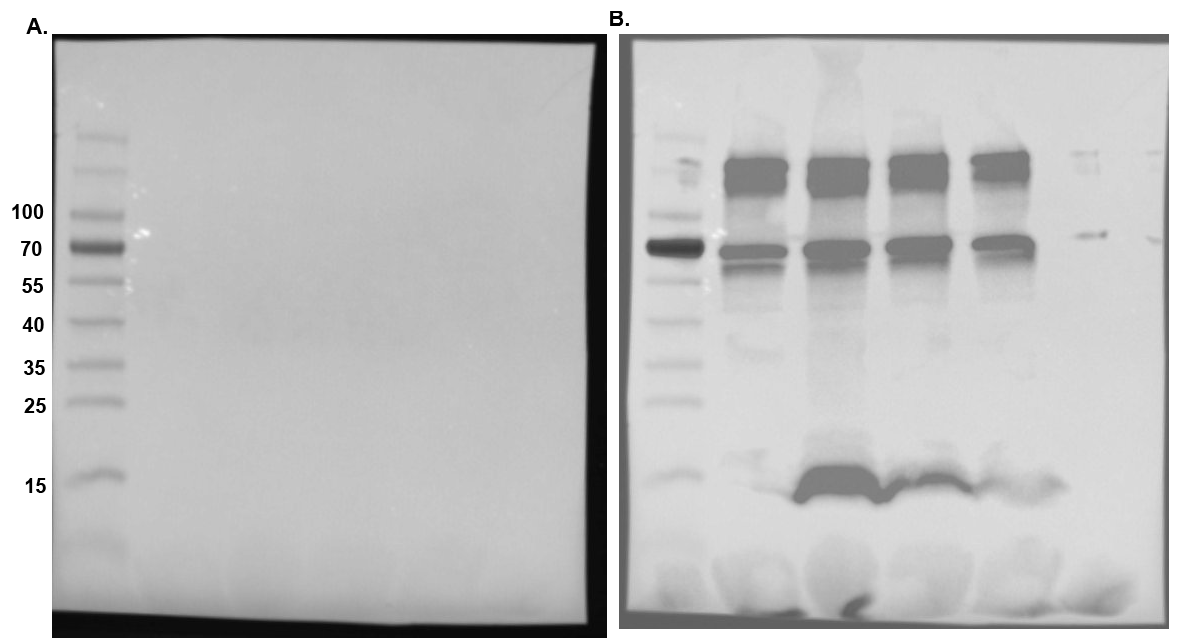


Figure S2: A) Development of marker under fluoroscence exposure in presence of EtBr to delineate markers clearly. B) Merged gel figures.
